# A Fragment

**Published:** 1918-11-30

**Authors:** 


### "A Fragment."

Watching and waiting upon the sick; giving of her energy, her sympathy to so full an extent to the poor sufferers in her ward, it seemed to the nurse as though her very soul had grown tired. Thoughts came no more to the tired brain, the body felt a machine, the mind had become numbed. Loneliness and deep depression had descended upon the night nurse.

It was during the small hours of the morning in a London hospital. Would the world always be full of sickness, sorrow, and disease? Must there *always* be nurses, and among them those who act cleverly but without heart: no encouraging word spoken by them to the patient, no *real* link of sympathy, no *real* giving of self which true nursing demands? Gazing through the ward window the nurse questioned the heavens. Far below sounded still some footsteps and rumbling of wheels. Suddenly, a chorus of music sounded on the night air through the open window, coming from no distinct direction, but flooding the night with sweet sounds. The nurse leant far out and listened, not questioning from whence the music came; it revived her soul and brought back her courage and high ideals. Life was to be lived, and in the service of others lies true happiness, was the thought which came to her with repeated insistence.

Closing the window when once more all was quiet she returned to her duties to give of herself again, knowing no stint in the giving. Into that woman's heart came the knowledge that the music was of no earthly making; her soul needed refreshment and courage; it had come in a way she would never have sought.

